# 1,4-Bis(pyrimidin-2-yl­sulfanyl)­butane

**DOI:** 10.1107/S1600536810041103

**Published:** 2010-10-20

**Authors:** Muhammad Akbar, Fahim Ashraf Quereshi, Waqar Nasir, Ahmad Adnan, Seik Weng Ng

**Affiliations:** a Department of Chemistry, Government College University, 54000 Lahore, Pakistan; b Department of Chemistry, University of Malaya, 50603 Kuala Lumpur, Malaysia

## Abstract

The –SCH_2_CH_2_CH_2_CH_2_S– portion of the title compound, C_12_H_14_N_2_S_2_, adopts an extended zigzag conformation. The angles at the tetra­hedral carbon atoms are marginally increased [113.63 (12)° and 111.38 (17)° for S—C—C and C—C—C respectively] from the idealized tetra­hedral angle. The mol­ecule lies on an inversion center located at the mid-point of the butyl chain. In the crystal, there is a π–π stacking inter­action between inversion-related pyrimidine rings with mean inter­planar spacing of 3.494 (2) Å.

## Related literature

For the structure of a silver perchlorate adduct of the title compound see: Wang & Zheng (2007 ▶).
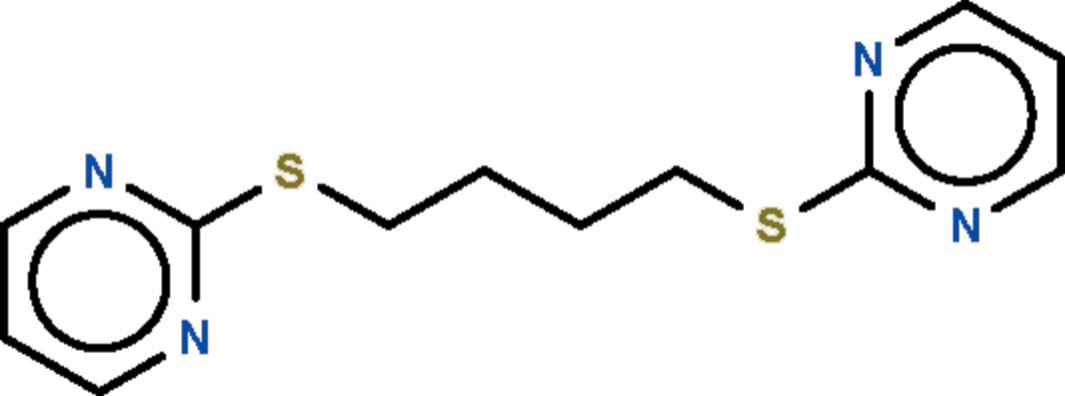


## Experimental

### 

#### Crystal data

C_12_H_14_N_4_S_2_
*M* *_r_* = 278.39
Triclinic, 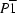

*a* = 5.5025 (1) Å
*b* = 7.6617 (1) Å
*c* = 8.3598 (2) Å
α = 86.915 (1)°
β = 87.253 (1)°
γ = 75.853 (1)°
*V* = 341.03 (1) Å^3^
*Z* = 1
Mo *K*α radiation
μ = 0.38 mm^−1^
*T* = 293 K
0.35 × 0.20 × 0.10 mm

#### Data collection

Bruker Kappa APEXII diffractometer
Absorption correction: multi-scan (*SADABS*; Sheldrick, 1996 ▶) *T*_min_ = 0.849, *T*_max_ = 1.000
5571 measured reflections
1524 independent reflections
1384 reflections with *I* > 2σ(*I*)
*R*_int_ = 0.025

#### Refinement

*R*[*F* ^2^ > 2σ(*F* ^2^)] = 0.036
*wR*(*F* ^2^) = 0.115
*S* = 1.05
1524 reflections
82 parameters
H-atom parameters constrained
Δρ_max_ = 0.23 e Å^−3^
Δρ_min_ = −0.23 e Å^−3^

### 

Data collection: *APEX2* (Bruker, 2009 ▶); cell refinement: *SAINT* (Bruker, 2009 ▶); data reduction: *SAINT*; program(s) used to solve structure: *SHELXS97* (Sheldrick, 2008 ▶); program(s) used to refine structure: *SHELXL97* (Sheldrick, 2008 ▶); molecular graphics: *X-SEED* (Barbour, 2001 ▶); software used to prepare material for publication: *publCIF* (Westrip, 2010 ▶).

## Supplementary Material

Crystal structure: contains datablocks global, I. DOI: 10.1107/S1600536810041103/pk2276sup1.cif

Structure factors: contains datablocks I. DOI: 10.1107/S1600536810041103/pk2276Isup2.hkl

Additional supplementary materials:  crystallographic information; 3D view; checkCIF report

## Figures and Tables

**Fig. 1.**
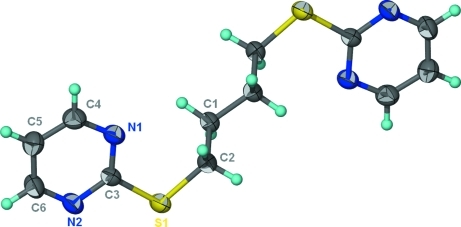
Thermal ellipsoid plot (Barbour, 2001) of C12H14N2S2 at the 50% probability level; hydrogen atoms are drawn as spheres of arbitrary radius. The molecule lies about a center-of-inversion.

## supplementary crystallographic information

### Comment

The bis(arylthio)alkane ligands are excellent 'flexible' ligands for binding to silver(I) compounds. The title ligand (Scheme I) has been used in the synthesis of a silver perchlorate adduct; the ligand binds through its nitrogen donor sites (Wang & Zheng, 2007). The ligand itself exists as a centrosymmetric compound (Fig. 1) with an inversion center located at the mid-point of the butyl chain.  The –SCH_2_CH_2_CH_2_CH_2_S– portion of the molecule of C_12_H_14_N_2_S_2_ adopts an extended zigzag conformation, and the angles at the tetrahedral C atoms are marginally increased from the idealized 109.5 ° (113.62 (12)° and 111.38 (17)° for S—C—C and C—C—C respectively).

### Experimental

To the ethanol mixture (50 ml) of 2-mercaptopyrimidine (2 g, 17.8 mmol) and sodium bicarbonate (1.8 g, 21.4 mmol) was added 1,4-dichlorobutane (1.13 g, 8.92 mmol). The mixture was heated for 6 h and the progress of the reaction was monitored by TLC (chloroform: ethyl acetate 9:1). The mixture was filtered and the solvent was allowed to evaporate. The colorless crystals that were isolated were collected and washed with hexane; yield 82%.

### Refinement

Carbon-bound H-atoms were placed in calculated positions (C–H 0.93 to 0.97 Å) and were included in the refinement in the riding model approximation, with *U*(H) set to 1.2*U*(C).

### Crystal data

**Table:**

C_12_H_14_N_4_S_2_	*Z* = 1
*M**_r_* = 278.39	*F*(000) = 146
Triclinic, *P*1	*D*_x_ = 1.356 Mg m^−^^3^
Hall symbol: -P 1	Mo *K*α radiation, λ = 0.71073 Å
*a* = 5.5025 (1) Å	Cell parameters from 3569 reflections
*b* = 7.6617 (1) Å	θ = 2.4–28.3°
*c* = 8.3598 (2) Å	µ = 0.38 mm^−^^1^
α = 86.915 (1)°	*T* = 293 K
β = 87.253 (1)°	Prism, pale yellow
γ = 75.853 (1)°	0.35 × 0.20 × 0.10 mm
*V* = 341.03 (1) Å^3^	

### Data collection

**Table:**

Bruker Kappa APEXII diffractometer	1524 independent reflections
Radiation source: fine-focus sealed tube	1384 reflections with *I* > 2σ(*I*)
graphite	*R*_int_ = 0.025
Detector resolution: 0 pixels mm^-1^	θ_max_ = 27.5°, θ_min_ = 2.7°
φ and ω scans	*h* = −7→7
Absorption correction: multi-scan (*SADABS*; Sheldrick, 1996)	*k* = −9→9
*T*_min_ = 0.849, *T*_max_ = 1.000	*l* = −10→10
5571 measured reflections	

### Refinement

**Table:**

Refinement on *F*^2^	Primary atom site location: structure-invariant direct methods
Least-squares matrix: full	Secondary atom site location: difference Fourier map
*R*[*F*^2^ > 2σ(*F*^2^)] = 0.036	Hydrogen site location: inferred from neighbouring sites
*wR*(*F*^2^) = 0.115	H-atom parameters constrained
*S* = 1.05	*w* = 1/[σ^2^(*F*_o_^2^) + (0.0684*P*)^2^ + 0.0791*P*] where *P* = (*F*_o_^2^ + 2*F*_c_^2^)/3
1524 reflections	(Δ/σ)_max_ = 0.001
82 parameters	Δρ_max_ = 0.23 e Å^−^^3^
0 restraints	Δρ_min_ = −0.23 e Å^−^^3^

### Fractional atomic coordinates and isotropic or equivalent isotropic displacement parameters (Å^2^)

**Table:**

	*x*	*y*	*z*	*U*_iso_*/*U*_eq_	
S1	0.00757 (8)	0.83807 (5)	0.36197 (5)	0.0497 (2)	
N1	0.2131 (3)	0.68013 (19)	0.09155 (18)	0.0464 (3)	
N2	−0.2078 (3)	0.8589 (2)	0.09199 (19)	0.0505 (4)	
C1	0.3698 (3)	0.5370 (2)	0.46719 (19)	0.0437 (4)	
H1A	0.3522	0.4713	0.3742	0.052*	
H1B	0.2444	0.5191	0.5479	0.052*	
C2	0.3247 (3)	0.7362 (2)	0.4204 (2)	0.0469 (4)	
H2A	0.4395	0.7516	0.3321	0.056*	
H2B	0.3622	0.7989	0.5103	0.056*	
C3	0.0099 (3)	0.78350 (19)	0.16033 (19)	0.0396 (3)	
C4	0.1936 (3)	0.6518 (3)	−0.0632 (2)	0.0533 (4)	
H4	0.3305	0.5800	−0.1171	0.064*	
C5	−0.0195 (4)	0.7241 (3)	−0.1458 (2)	0.0541 (4)	
H5	−0.0289	0.7042	−0.2539	0.065*	
C6	−0.2187 (3)	0.8274 (3)	−0.0615 (2)	0.0539 (4)	
H6	−0.3667	0.8770	−0.1140	0.065*	

### Atomic displacement parameters (Å^2^)

**Table:**

	*U*^11^	*U*^22^	*U*^33^	*U*^12^	*U*^13^	*U*^23^
S1	0.0475 (3)	0.0499 (3)	0.0434 (3)	0.00536 (19)	−0.00486 (18)	−0.00421 (18)
N1	0.0412 (7)	0.0462 (7)	0.0472 (8)	−0.0017 (6)	−0.0016 (6)	−0.0023 (6)
N2	0.0391 (7)	0.0549 (8)	0.0517 (9)	0.0004 (6)	−0.0063 (6)	−0.0012 (6)
C1	0.0419 (8)	0.0443 (8)	0.0420 (8)	−0.0037 (6)	−0.0078 (7)	−0.0004 (6)
C2	0.0467 (8)	0.0440 (8)	0.0470 (9)	−0.0035 (7)	−0.0116 (7)	−0.0020 (7)
C3	0.0384 (7)	0.0352 (7)	0.0428 (8)	−0.0048 (6)	−0.0032 (6)	0.0013 (6)
C4	0.0500 (10)	0.0566 (10)	0.0485 (10)	−0.0038 (8)	0.0045 (7)	−0.0067 (8)
C5	0.0603 (11)	0.0596 (10)	0.0419 (9)	−0.0130 (8)	−0.0047 (8)	−0.0021 (7)
C6	0.0474 (9)	0.0605 (10)	0.0514 (10)	−0.0075 (8)	−0.0134 (8)	0.0033 (8)

### Geometric parameters (Å, °)

**Table:**

S1—C3	1.7571 (17)	C1—H1B	0.9700
S1—C2	1.8076 (16)	C2—H2A	0.9700
N1—C3	1.328 (2)	C2—H2B	0.9700
N1—C4	1.337 (2)	C4—C5	1.370 (3)
N2—C6	1.325 (2)	C4—H4	0.9300
N2—C3	1.337 (2)	C5—C6	1.374 (3)
C1—C2	1.517 (2)	C5—H5	0.9300
C1—C1^i^	1.524 (3)	C6—H6	0.9300
C1—H1A	0.9700		
			
C3—S1—C2	103.41 (8)	H2A—C2—H2B	107.7
C3—N1—C4	115.09 (14)	N1—C3—N2	127.07 (15)
C6—N2—C3	115.78 (15)	N1—C3—S1	120.73 (12)
C2—C1—C1^i^	111.38 (17)	N2—C3—S1	112.20 (12)
C2—C1—H1A	109.4	N1—C4—C5	122.81 (16)
C1^i^—C1—H1A	109.4	N1—C4—H4	118.6
C2—C1—H1B	109.4	C5—C4—H4	118.6
C1^i^—C1—H1B	109.4	C4—C5—C6	116.83 (17)
H1A—C1—H1B	108.0	C4—C5—H5	121.6
C1—C2—S1	113.63 (12)	C6—C5—H5	121.6
C1—C2—H2A	108.8	N2—C6—C5	122.41 (16)
S1—C2—H2A	108.8	N2—C6—H6	118.8
C1—C2—H2B	108.8	C5—C6—H6	118.8
S1—C2—H2B	108.8		
			
C1^i^—C1—C2—S1	−173.69 (15)	C2—S1—C3—N1	3.47 (15)
C3—S1—C2—C1	−82.52 (14)	C2—S1—C3—N2	−175.76 (12)
C4—N1—C3—N2	0.6 (3)	C3—N1—C4—C5	0.2 (3)
C4—N1—C3—S1	−178.46 (12)	N1—C4—C5—C6	−0.8 (3)
C6—N2—C3—N1	−0.7 (3)	C3—N2—C6—C5	−0.1 (3)
C6—N2—C3—S1	178.47 (13)	C4—C5—C6—N2	0.8 (3)

Symmetry codes: (i) −*x*+1, −*y*+1, −*z*+1.
